# Roles of STAT3 and STAT Family Proteins and Their Signaling Pathways in Thyroid Cancer

**DOI:** 10.3390/cells15100884

**Published:** 2026-05-12

**Authors:** Chie Masaki, Norihito Inoue, Tomohiro Chiba

**Affiliations:** 1 Department of Pathology, Cancer Institute Hospital of Japanese Foundation for Cancer Research, Tokyo 135-8550, Japan; c-masaki@ito-hospital.jp (C.M.); norihito.inoue@jfcr.or.jp (N.I.); 2 Department of Surgery, Ito Hospital, Tokyo 150-8308, Japan; 3 Department of Cytology, Cancer Institute Hospital of Japanese Foundation for Cancer Research, Tokyo 135-8550, Japan

**Keywords:** thyroid cancer, STAT3, JAK-STAT pathway, targeted therapy, drug resistance, tumor microenvironment, double-edged sword, BRAF inhibitor

## Abstract

**Highlights:**

**What are the main findings?**

- STAT3 acts as a “double-edged sword” in thyroid cancer: hyperactive STAT3 drives metastasis and BRAF inhibitor resistance in advanced carcinomas, yet paradoxically acts as a tumor suppressor by restraining the Warburg effect via non-canonical mitochondrial localization.
- Clinically, preserved nuclear STAT3 independently predicts a favorable prognosis and is inversely correlated with *TERT* promoter mutations, offering a biological modifier for clinical risk stratification.

**What are the implications of the main findings?**

- Evaluating nuclear STAT3 expression can serve as a vital biological modifier for refining and complementing clinical risk stratification frameworks.
- While novel direct STAT3 inhibitors and rational combinations with immune checkpoint inhibitors or STING agonists promise to overcome refractory diseases, STATs’ intricate dual functionality demands rigorous biomarker-guided precision medicine.

**Abstract:**

Signal transducers and activators of transcription (STAT) proteins, which operate via canonical and non-canonical mechanisms, are critically implicated in thyroid tumorigenesis. This review integrates their multifaceted roles in thyroid cancer. STAT3 acts as a “double-edged sword”: hyperactive STAT3 drives metastasis and BRAF inhibitor resistance in advanced carcinomas, yet paradoxically acts as a tumor suppressor by restraining the Warburg effect via non-canonical mitochondrial localization. Clinically, preserved nuclear STAT3 independently predicts a favorable prognosis and is inversely correlated with *TERT* promoter mutations, offering a biological modifier for clinical risk stratification. Furthermore, STAT1 regulates differentiation via the IGF2BP2-*m^6^A* axis, STAT5 drives proliferation upon release from TRβ suppression, and STAT6 confers chemoresistance. While novel direct STAT3 inhibitors (e.g., TTI-101) and rational combinations with immune checkpoint inhibitors or STING agonists show promise in overcoming refractory disease, the intricate dual functionality of STAT family proteins demands rigorous biomarker-guided precision medicine approaches.

## 1. Introduction

Thyroid cancer is the most common malignancy of the endocrine system, with incidence rates that have increased significantly in recent decades [1,2]. While the majority of thyroid cancers are differentiated thyroid carcinomas (DTC), including papillary thyroid carcinoma (PTC) and follicular thyroid carcinoma (FTC), with generally favorable prognoses, a subset of patients develops aggressive forms, such as poorly differentiated thyroid carcinoma (PDTC) and anaplastic thyroid carcinoma (ATC) [3,4]. The pathogenesis of thyroid cancer is frequently driven by genetic alterations in the MAPK and PI3K/AKT signaling pathways, most notably the *BRAF* p.V600E mutation, *RAS* mutations, and *RET/PTC* rearrangements [3,4,5,6,7,8,9]. Although the recently updated 2025 American Thyroid Association (ATA) guidelines provide a refined risk stratification framework incorporating clinicopathological features and targeted molecular testing (e.g., *BRAF* and *TERT* mutations), this anatomically driven system may not fully capture the dynamic intratumoral biology and metabolic reprogramming that ultimately dictate tumor recurrence [5,6].

Beyond these classical drivers, the Signal Transducer and Activator of Transcription (STAT) family of proteins, particularly STAT3, has emerged as a critical node in thyroid tumorigenesis [10]. STAT proteins serve as the primary downstream transcription factors of the canonical Janus kinase (JAK)/STAT (JAK/STAT) signaling cascade, mediating essential physiological responses to cytokines and growth factors. While STAT proteins mediate essential physiological responses to cytokines and growth factors, their dysregulation is implicated in cancer progression. Among them, STAT3 is widely recognized as a potent oncogene that promotes cell proliferation, survival, angiogenesis, and immune evasion [11]. In advanced thyroid cancers, hyperactive STAT3 signaling drives epithelial–mesenchymal transition (EMT), cancer stem cell maintenance, and acquired resistance to standard targeted therapies, such as BRAF inhibitors [12,13]. However, the role of STAT3 in thyroid cancer is complex and context-dependent. Beyond its canonical nuclear functions, STAT3 is involved in non-canonical pathways, most notably in the regulation of cellular energy metabolism. A growing body of preclinical and clinical evidence has revealed that STAT3 paradoxically functions as a robust tumor suppressor by restraining metabolic processes, suppressing the Warburg effect, and preventing rapid tumor expansion in certain genetic contexts [14].

This review provides a comprehensive overview of the STAT family proteins in thyroid cancer, emphasizing the “double-edged sword” nature of STAT3. By synthesizing recent molecular insights and clinical correlations, we highlight the prognostic value of STAT3 alongside evolving risk paradigms, explore its crosstalk with other signaling pathways, and discuss the precision medicine considerations required for the safe targeting of the JAK/STAT axis in refractory thyroid malignancies.

## 2. STAT Family Proteins and Their Signaling Pathways: Overview

### 2.1. Domain Architecture and Canonical Activation

**Figure 1:**
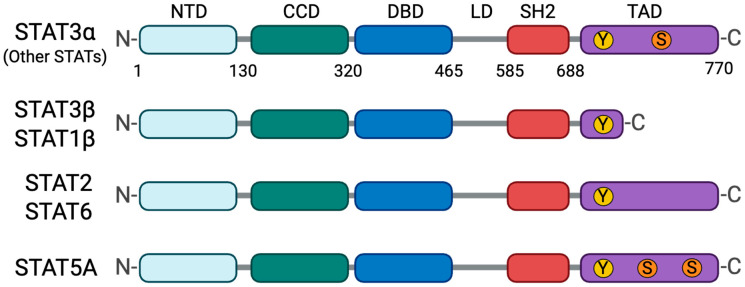
Structure of STAT Family Proteins. Schematic representation of the structurally conserved modular architecture of the STAT proteins. The six functional domains, namely the amino-terminal domain (NTD), coiled-coil domain (CCD), DNA-binding domain (DBD), linker domain (LD), Src homology 2 (SH2) domain, and carboxyl-terminal transactivation domain (TAD), are depicted. The tyrosine phosphorylation sites in the TAD, indicated as “Y” in yellow circles, are essential for STAT activation. There are some variations in the structures of STAT family proteins. Through alternative splicing, STAT3 and STAT1 produce full-length α isoforms and truncated β isoforms lacking a TAD. The α isoforms drive the canonical signaling pathway, whereas the β isoforms have distinct functional roles. STAT2 and STAT6 lack serine phosphorylation sites, indicated as “S” in orange circles, in their TAD. STAT5A has two serine phosphorylation sites in the TAD.

The STAT protein family consists of seven members in mammals: STAT1, STAT2, STAT3, STAT4, STAT5A, STAT5B, and STAT6, which function as latent cytoplasmic transcription factors mediating cellular responses to cytokines, growth factors, and interferons. All STAT family members share a structurally conserved modular architecture consisting of six domains (Figure 1) [15,16,17]. The N-terminal domain (NTD) facilitates dimer formation and cooperative binding to tandem DNA elements. The coiled-coil domain (CCD) is primarily responsible for receptor docking and interaction with regulatory proteins. The DNA-binding domain (DBD) makes sequence-specific contacts with consensus sequences, such as the gamma-activated site (GAS) or interferon-stimulated response elements (ISRE), whereas the linker domain (LD) transmits allosteric communication across the molecule. The highly conserved Src homology 2 (SH2) domain serves a functionally pivotal dual role: it first docks the latent STAT monomer to phosphorylated tyrosine motifs on the receptor and subsequently anchors two phosphorylated STAT monomers together into an antiparallel dimer. Finally, the C-terminal transactivation domain (TAD) recruits coactivators and houses critical regulatory phosphorylation sites.

**Figure 2:**
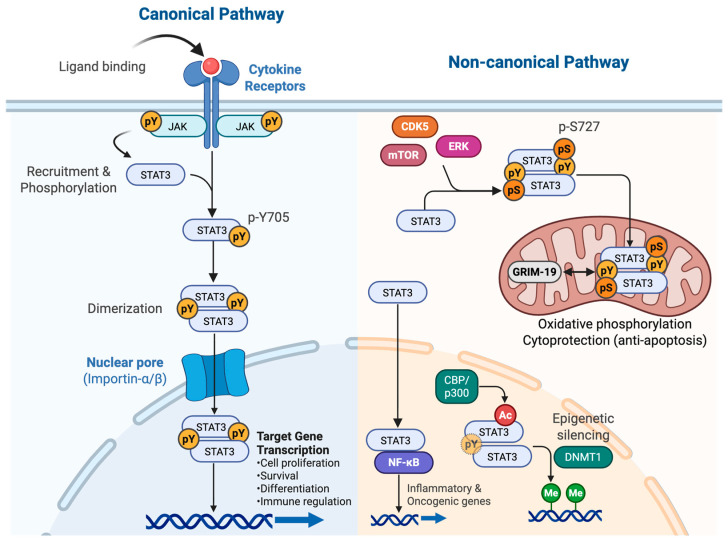
Canonical and non-canonical JAK/STAT signaling pathways. (**Left**) Canonical pathway: Cytokine or growth factor (ligand) binding induces receptor dimerization, bringing Janus kinases (JAKs) into proximity for transphosphorylation and activation. Activated JAKs phosphorylate receptor tyrosines and recruit STAT monomers via SH2 domains. JAK-mediated tyrosine phosphorylation, such as the phosphorylation of tyrosine 705 (Y705) in STAT3, indicated as “pY” in yellow circles, promotes dimerization and nuclear translocation through the importin-α/β complex, leading to the transcription of genes involved in proliferation, survival, differentiation, and immune regulation. (**Right**) Non-canonical pathways: Beyond canonical tyrosine phosphorylation, STAT3 activity is governed by various post-translational modifications (PTMs) that dictate distinct non-canonical roles. Serine phosphorylation at S727 (pS727), indicated as “pS” in orange circles, is induced by serine kinases such as CDK5, ERK, and mTOR, which drives a distinct pool of STAT3 into the mitochondria, where it interacts with GRIM-19 to maintain mitochondrial functions. CBP/p300-mediated acetylation at lysine 685 (K685), indicated as “Ac” in red circles, enables pY705-independent dimerization and recruits DNMT1 to epigenetically silence the tumor suppressor genes via promotor DNA methylation, indicated as “Me” in green circles. Unphosphorylated STAT3 (uSTAT3) can translocate to the nucleus to induce inflammatory and oncogenic genes in complex with NF-κB.

The canonical JAK/STAT activation pathway (Figure 2, left) is a highly ordered cascade that occurs rapidly upon stimulation [10]. The binding of a ligand (e.g., IL-6 family cytokines, interferons, or growth factors) induces receptor dimerization, bringing receptor-associated JAKs (JAK1, JAK2, JAK3, or TYK2) into close proximity. This proximity drives the transphosphorylation and activation of JAKs, which subsequently phosphorylate specific intracellular tyrosine residues on the receptor tail. Latent STAT monomers are recruited to these docking sites via their SH2 domains and are directly phosphorylated by JAKs on a critical carboxyl-terminal tyrosine residue (e.g., tyrosine 705 [Y705] in STAT3). Phosphorylated STATs then dissociate from the receptor, homodimerize or heterodimerize, and translocate into the nucleus via the importin-α/β complex. Once in the nucleus, STAT dimers bind to specific DNA elements to activate the transcription of target genes that govern proliferation, survival, angiogenesis, and differentiation.

### 2.2. Post-Translational Modifications and Non-Canonical Pathways

While tyrosine phosphorylation (i.e., pY705) is the master “on” switch for canonical signaling, STAT3 activity is governed by a rich landscape of over 80 post-translational modifications (PTMs) that dictate various non-canonical functions [18,19,20,21] (Figure 2, right). These PTMs include acetylation, methylation, and SUMOylation. They govern distinct non-canonical functions, alter subcellular localization, and shape the ultimate biological output. Specific physiological and microenvironmental factors in cancer cells, such as oxidative stress, metabolic shifts (e.g., hypoxia), and serine kinase activity (e.g., MAPKs), trigger non-canonical signaling by pushing STAT3 away from the canonical DNA-binding location in the nucleus.

#### 2.2.1. Serine Phosphorylation and Mitochondrial STAT3 (mitoSTAT3)

Phosphorylation of serine 727 (pS727) is mediated by kinases such as MAPK/ERK, CDK5, and mTOR. Although pS727 is required for maximal transcriptional activation in the nucleus, it is also the predominant modification found in a distinct pool of STAT3 localized to the inner mitochondrial membrane [22,23]. Independent of nuclear gene transcription, mitoSTAT3 interacts with GRIM-19 to support the activity of the electron transport chain (complexes I, II, and V), promoting oxidative phosphorylation (OXPHOS), and restraining Warburg-type aerobic glycolysis [22]. The coexistence of pro-growth nuclear STAT3 and anti-Warburg mitoSTAT3 illustrates a compartment-specific duality that is directly relevant to its tumor-suppressive role in the thyroid epithelium.

#### 2.2.2. Other Modifications: Acetylation, SUMOylation, Methylation and Unphosphorylated STAT3 (uSTAT3)

Acetylation is a highly dynamic and reversible modification governed by the opposing actions of histone acetyltransferases (HATs), such as CBP/p300, and histone deacetylases (HDACs) or sirtuins [24]. In the context of STAT3, acetylation regulates chromatin accessibility, protein–protein interactions, and dimer stability. The most extensively characterized acetylation site is Lysine 685 (K685), located in the SH2 domain [24]. Acetylation of K685 (Ac-K685) by CBP/p300 promotes STAT3 dimerization independent of pY705 [24]. Ac-K685-STAT3 also recruits DNA methyltransferase 1 (DNMT1) to silence various tumor suppressor genes, including *CDKN2A*, *STAT1*, *TP53*, and *SOCS3* [25]. Furthermore, acetylation at N-terminal residues, such as K49 and K87, stabilizes the interaction between STAT3 and p300, enhancing transcriptional transactivation [26]. Additionally, K87 acetylation facilitates the translocation of STAT3 into the mitochondria, linking this PTM directly to the metabolic regulation of tumor cells [27].

SUMOylation is the covalent attachment of Small Ubiquitin-like Modifier (SUMO) proteins to target lysines. This process is typically mediated by a cascade of enzymes, often involving protein inhibitor of activated STAT (PIAS) proteins functioning as SUMO E3 ligases, which generally act to negatively regulate STAT3 signaling or target the protein for degradation. The primary target of this modification is lysine 451 (K451) [28,29]. Modification of K451 by SUMO2/3 strongly promotes the interaction between STAT3 and the nuclear protein tyrosine phosphatase TC45, a splice variant of T-cell protein tyrosine phosphatase, enhancing its dephosphorylation and terminating canonical signaling [26]. This SUMO-dependent recruitment of TC45 facilitates the rapid dephosphorylation of pY705, effectively terminating canonical STAT3 transcriptional activity and triggering its export from the nucleus. Conversely, de-SUMOylation of this residue by enzymes, such as SUMO-specific peptidase 3 (SENP3), sustains STAT3 hyperphosphorylation and prolongs activation in the tumor microenvironment [28].

Protein methylation involves the transfer of methyl groups to specific amino acid residues, catalyzed by histone methyltransferases, and is reversible via demethylases. For STAT3, methylation generally occurs on specific lysine residues within the N-terminal and coiled-coil domains, serving as either positive or negative regulators of gene transcription [30]. Methylation of K49 and K180 is mediated by the Enhancer of Zeste Homolog 2 (EZH2). Dimethylation at K49 profoundly enhances the expression of IL-6-induced target genes, whereas K180 methylation actively promotes and protects pY705, driving a potent pro-tumorigenic signal often observed in aggressive cancers, such as glioblastoma [31,32]. Conversely, dimethylation of K140 by SET domain containing 9 (SET9) methyltransferase has an inhibitory effect. K140 methylation occurs inside the nucleus (requiring prior pS727) and acts as a transcriptional repressor by impeding STAT3’s ability to bind to DNA [30].

Historically considered a latent, inactive precursor, unphosphorylated STAT3 (uSTAT3) is now recognized to possess distinct biological functions [33]. uSTAT3 can translocate from the cytoplasm to the nucleus independently of pY705, utilizing importin-α3 rather than the classical importin-α5/β1 complex [34]. Once in the nucleus, uSTAT3 regulates transcription through novel mechanisms, frequently by forming heterodimers with other transcription factors, such as unphosphorylated NF-κB. This uSTAT3/NF-κB complex binds to distinct promoter regions to drive a second, prolonged wave of expression of highly oncogenic and inflammatory genes (e.g., *IL-6*, *IL-8*, and *RANTES*) [35]. Conversely, uSTATs can associate with heterochromatin protein 1 (HP1) to stabilize heterochromatin and protect against genomic instability [36].

### 2.3. Transcriptional and Post-Transcriptional Regulation of STAT3

Aberrant hyperactivation of STAT3 in cancer is not solely driven by continuous upstream kinase signaling; it is also profoundly dictated by intrinsic transcriptional controls and a complex network of post-transcriptional regulators, including microRNAs (miRNAs) and long non-coding RNAs (lncRNAs).

#### 2.3.1. Transcriptional Regulation of *STAT3*

At the transcriptional level, *STAT3* is regulated by several oncogenic transcription factors and feedback loops. Notably, STAT3 tightly regulates its own expression. Phosphorylated STAT3 (pSTAT3) directly binds to the STAT3 promoter to activate its own transcription, creating a positive feedback loop that generates de novo uSTAT3 molecules [37]. Over time, this autoregulation significantly elevates the total intracellular STAT3 pool, supporting prolonged transcriptional responses. Furthermore, other oncogenic transcription factors directly upregulate *STAT3* expression. In ATC, for example, the gain-of-function mutant *TP53* acts as a potent transcriptional activator of *STAT3*. This mutant *TP53*-driven upregulation of STAT3 provides a critical anti-apoptotic survival mechanism for aggressive thyroid cancer cells [38,39].

#### 2.3.2. Post-Transcriptional Regulation by Non-Coding RNAs

Post-transcriptional regulation by non-coding RNAs adds another critical layer of complexity to STAT3 activation. A vast array of miRNAs directly target the 3′-untranslated region (3′-UTR) of the *STAT3* mRNA to inhibit its translation. Tumor-suppressive miRNAs, such as miR-125b-5p, members of the Let-7 family, miR-15a, miR-17-5p, and miR-20a, directly downregulate *STAT3* expression; their frequent silencing in cancer removes this post-transcriptional brake, permitting STAT3 accumulation [40,41,42,43].

Conversely, miRNAs can indirectly hyperactivate STAT3 by targeting JAK/STAT pathway inhibitors. For instance, miR-19a, miR-30d, and miR-155 directly target and suppress *SOCS1* [44,45,46], whereas miR-18a specifically targets *PIAS3* [47]. The upregulation of these oncogenic miRNAs in the tumor microenvironment effectively dismantles the physiological negative feedback loops, leading to unchecked STAT3 signaling. Furthermore, STAT3 reciprocally regulates miRNAs; for example, STAT3 directly binds to the enhancer region of miR-21, driving its transcription and promoting oncogenesis [48,49].

lncRNAs also modulate STAT3 dynamically. LincRNA-p21 directly binds to STAT3 to inhibit its transcriptional activity, whereas the primate-specific lncRNA FLANC (flamingo non-coding RNA) upregulates and prolongs the half-life of pSTAT3 [50]. Additionally, lncRNA MEG3 (Maternally Expressed Gene 3) facilitates the ubiquitination and subsequent proteasomal degradation of STAT3, underscoring the RNA-guided regulation of STAT3 stability [51].

#### 2.3.3. Ubiquitin-Mediated Regulation and Epigenetic Crosstalk

While PTMs regulate STAT activity and localization, the ubiquitin (Ub)-proteasome system is a fundamental controller of JAK/STAT signal amplitude, protein stability, and therapeutic responsiveness. Ub-mediated regulation is integral to maintaining cellular homeostasis, and its dysregulation is a hallmark of tumorigenic plasticity.

Crucially, the regulation of JAK/STAT signaling is intimately shaped by crosstalk between Ub ligases and chromatin modifiers. HDAC1 and HDAC2 tightly regulate mutation-driven JAK-STAT signaling through the Ub ligase SIAH2 [52]. Global transcriptomic profiling identified the JAK/STAT pathway as the primary SIAH2-dependent target. Interestingly, these studies revealed a tumor-context-specific duality in SIAH2 function; for instance, the targeted knockout of SIAH2 renders cells markedly less sensitive to the cytotoxic effects of HDAC inhibitors. This demonstrates that Ub ligases not only degrade signaling components but also fundamentally control the epigenetic state and therapeutic vulnerability of tumors. Consequently, understanding the intersection of HDAC biology, Ub ligases such as SIAH2, and JAK/STAT signal modulation is essential for developing rational combinatorial strategies to overcome resistance in aggressive malignancies.

### 2.4. Negative Regulation of JAK/STAT Signaling

**Figure 3:**
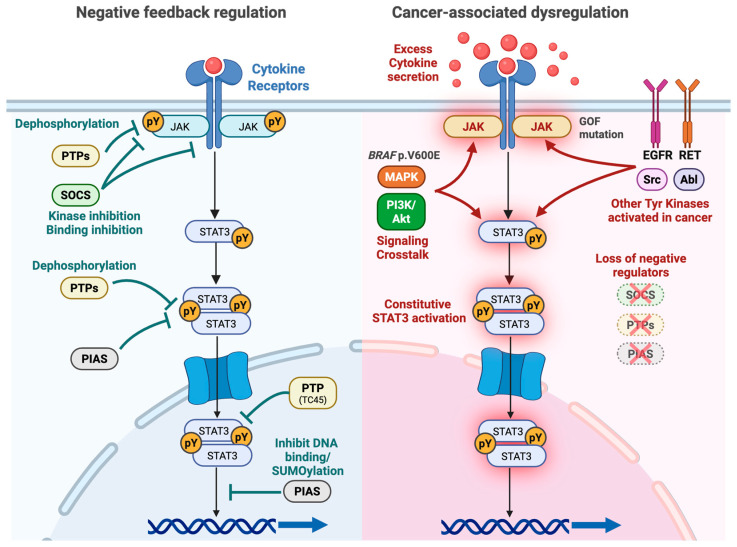
Negative Regulation and cancer-associated dysregulation of the JAK/STAT Signaling Pathway. (**Left**) The JAK/STAT signaling cascade is tightly controlled by three major intracellular brake systems, namely the Suppressor of Cytokine Signaling (SOCS) family, Protein Inhibitor of Activated STAT (PIAS) proteins, and protein tyrosine phosphatases (PTPs), to prevent hyperactivation of the signals through a classic negative feedback loop. SOCS proteins, such as SOCS1 and SOCS3, competitively bind to receptors via their SH2 domains and directly inhibit JAK catalytic activity. PIAS proteins, such as PIAS3, specifically interact with activated STAT dimers in the nucleus to inhibit DNA binding and promote SUMOylation. PTPs, such as SHP-1 and SHP-2, dephosphorylate receptor docking sites in the cytoplasm, whereas TC45 dephosphorylates STATs in the nucleus. Phosphorylated tyrosine residues are indicated as “pY.” (**Right**) In cancer cells, including thyroid cancer cells, the JAK/STAT pathway can be activated by excessive cytokine secretion (indicated as red balls), constitutive activation of tyrosine kinases such as EGFR, RET, Src, and Abl, crosstalk with the PI3K/Akt and MAPK pathways, which are constitutively activated by oncogenic driver mutations such as *BRAF* p.V6000E, and loss of negative regulators due to epigenetic silencing that disrupts negative feedback loops.

Under physiological conditions, JAK/STAT signaling is tightly regulated by three major intracellular brake systems to prevent sustained hyperactive signaling (Figure 3, right panel). Key regulators include the Suppressor of Cytokine Signaling (SOCS) family, PIAS proteins, and protein tyrosine phosphatases (PTPs) [18].

SOCS family members, particularly SOCS1 and SOCS3, act through a classic negative feedback loop. They competitively bind to phosphorylated tyrosine docking sites on receptors (e.g., gp130) via their SH2 domains and directly inhibit JAK catalytic activity through their kinase inhibitory region (KIR) [53,54].

PIAS proteins interact specifically with dimeric activated STATs, inhibiting STAT activity by physically blocking DNA-binding, recruiting corepressor proteins, and promoting SUMOylation [55,56].

Multiple PTPs regulate the pathway at different subcellular locations. Cytoplasmic phosphatases such as SHP-1 and SHP-2 dephosphorylate receptor docking sites, whereas nuclear TC45 dephosphorylates STAT1 and STAT3 to terminate transcriptional activity [57].

In thyroid and other cancers, aberrant constitutive activation of the pathway is driven by multiple mechanisms (Figure 3, left). Hyperactivation is frequently triggered by excessive autocrine or paracrine secretion of cytokines, such as IL-6, IL-4, and IL-10, and growth factors, alongside constitutively active non-receptor and receptor tyrosine kinases, including Src, Abl, EGFR, and RET. Additionally, excessive signaling crosstalk with the PI3K/Akt and MAPK pathways further reinforces STAT3 activation. Furthermore, the loss of intrinsic negative regulators plays a pivotal role in sustaining the oncogenic cascade. In thyroid and other cancers, epigenetic silencing of these crucial regulators, notably through the hypermethylation of the *SOCS3* promoter [58,59], as well as the loss of PIAS3 and PTPs, profoundly disrupts the physiological feedback loops. Ultimately, this enables thyroid cancer cells to escape negative regulation, driving persistent and constitutive STAT3 activation that promotes tumor survival, progression, and therapy resistance.

### 2.5. Aberrant Activation of JAK/STAT Signaling

In cancer, the aberrant constitutive activation of JAK/STAT signaling is frequently triggered by excessive autocrine or paracrine secretion of inflammatory cytokines (e.g., IL-6) and constitutively active oncogenic kinases (Figure 3, right). However, genetic mutations are a critical mechanism of pathway hyperactivation that must be considered. As comprehensively outlined in a recent review by Mustafa & Krämer, the intersection of aberrant cytokine signaling, post-translational regulation, chromatin state, and specific JAK mutations generates profound pathway plasticity and distinct therapeutic vulnerabilities [60]. While primary activating mutations, such as *JAK2* p.V617F, are hallmarks of myeloproliferative neoplasms, mutational activation is increasingly recognized in solid tumors. In thyroid cancer, genomic and transcriptomic studies have revealed that tumors that dedifferentiate and acquire resistance to BRAF inhibitors can harbor newly acquired functional missense mutations in *JAK*1 and *JAK2*. This mutational pathway plasticity provides a bypass mechanism that sustains STAT activation, despite the pharmacological blockade of upstream MAPK drivers.

## 3. STAT3 in Thyroid Cancer: A Double-Edged Sword?

The function of STAT3 in thyroid cancer is exceptionally complex and context-dependent, with compelling preclinical and clinical evidence supporting both oncogenic and tumor-suppressive roles, depending on the specific genetic background and tumor microenvironment (TME).

### 3.1. STAT3 as an Oncogene

**Figure 4:**
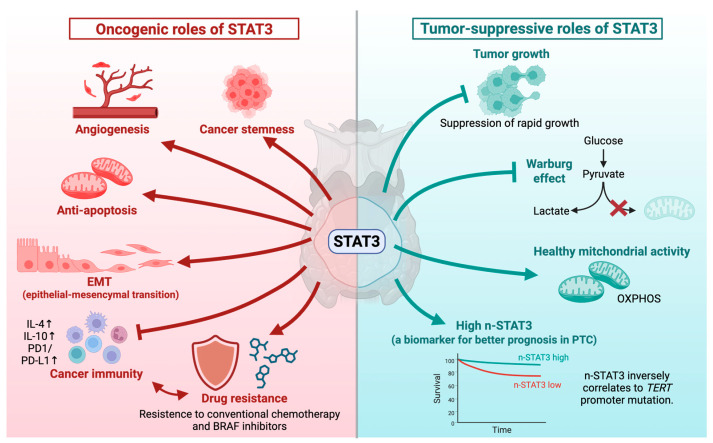
The “Double-Edged Sword” Role of STAT3 in Thyroid Cancer. (**Left**) Hyperactive STAT3 signaling drives aggressive tumor phenotypes through multiple molecular mechanisms. In aggressive thyroid carcinomas, STAT3 signaling is essential for maintaining cancer stemness, angiogenesis, and executing antiapoptotic functions (red arrows for induction). STAT3 activation also induces epithelial–mesenchymal transition (EMT). Furthermore, autocrine IL-4 and IL-10 loops constitutively activate the JAK/STAT and PI3K/AKT pathways to suppress cancer immunity, conferring resistance to chemotherapy (red T-bars for suppression). In addition, STAT3 mediates acquired resistance to targeted therapies, such as BRAF inhibitors. (**Right**) Paradoxically, in specific genetic and microenvironmental contexts, STAT3 robustly restrains thyroid tumor growth (green T-bars for suppression). Mechanistically, STAT3 acts as a vital metabolic checkpoint that suppresses aerobic glycolysis (the Warburg effect) in cancer cells. STAT3, especially mitochondrial STAT3, supports mitochondrial functions such as oxidative phosphorylation (OXPHOS) (green arrow for induction). Current clinical evidence shows that nuclear STAT3 (n-STAT3) expression is associated with a better prognosis in papillary thyroid carcinoma (PTC). The expression levels of n-STAT3 were inversely correlated with *TERT* promoter mutations.

A substantial body of evidence supports the oncogenic role of STAT3 in driving aggressive thyroid cancer phenotypes. High expression of phosphorylated STAT3 (pSTAT3) is frequently detected in PTC and ATC tissues compared to that in normal thyroid tissues [12,14,61,62]. The activation of STAT3 is significantly associated with epithelial–mesenchymal transition (EMT) (Figure 4, left), characterized by the downregulation of E-cadherin and upregulation of vimentin, thereby promoting tumor invasion and lymph node metastasis [18,63,64].

In the context of obesity-associated thyroid cancer, a high-fat diet in *ThrbPV/PVPten+/−* mice hyperactivates the leptin-JAK2-STAT3 signaling axis, promoting aggressive tumor growth, anaplasia, and reduced survival [65]. Targeted inhibition of STAT3 using S3I-201 in this model effectively delayed tumor progression, suppressed the expression of cell cycle regulators (Cyclin D1, Cyclin B1, CDK4, CDK6), and blocked EMT effectors, such as vimentin and MMP-2 [66,67].

In aggressive ATC, STAT3 is crucial for maintaining cancer stem cell-like properties and preventing cell death. A gain-of-function mutant *TP53* (e.g., p.G199V) found in ATC relies heavily on STAT3 signaling to exert potent anti-apoptotic functions; knockdown of this mutant *TP53* leads to downregulation of STAT3 and induction of apoptosis [38]. Furthermore, autocrine production of IL-4 and IL-10 by thyroid cancer cells constitutively activates the JAK/STAT and PI3K/AKT pathways, leading to the upregulation of anti-apoptotic molecules and conferring profound resistance to conventional chemotherapy [12,28,68].

STAT3 signaling is also deeply implicated in acquired resistance to targeted therapies. In *BRAF* p.V600E-mutated thyroid carcinoma cells, exposure to the BRAF inhibitor vemurafenib strongly upregulates an autocrine IL-6/STAT3 feed-forward loop that reactivates survival signals, impairing the cytostatic activity of the drug. Dual blockade of BRAF and the IL-6/STAT3 axis significantly improves the inhibition of cell cycle progression and overcomes resistance to BRAF-targeted therapy [69,70].

### 3.2. STAT3 as a Tumor Suppressor

Conversely, paradoxical findings have established that STAT3 robustly restrains tumor growth in specific thyroid cancer contexts. Couto et al. demonstrated that knocking down *STAT3* using shRNA in thyroid cancer cell lines did not affect in vitro proliferation but generated significantly larger and more invasive tumors in mouse xenograft models [14] (Figure 4, right). Similarly, thyrocyte-specific deletion of *STAT3* in a *BRAF* p.V600E-driven mouse model of PTC resulted in tumors that were larger, more proliferative, and exhibited early signs of aggressive solid growth compared to *STAT3*-wildtype tumors.

Mechanistically, STAT3 functions as a critical metabolic checkpoint. STAT3-deficient thyroid tumors exhibit increased glucose consumption, enhanced lactate production, and upregulation of HIF1α target genes, suggesting that STAT3 suppresses aerobic glycolysis (the Warburg effect). Furthermore, genome-wide expression profiling identified the tumor suppressor *Insulin-Like Growth Factor Binding Protein 7* (*IGFBP7*) as a key downstream transcriptional target of STAT3. Loss of *STAT3* dramatically reduces *IGFBP7* expression, effectively removing critical brakes on tumor expansion [11].

### 3.3. Clinical Prognostic Significance of Nuclear STAT3 Expression

The clinical relevance of STAT3’s tumor-suppressive activity was recently validated in a large retrospective study of 1132 PTC cases [71]. This study revealed that nuclear STAT3 (n-STAT3) expression is an independent and favorable prognostic biomarker. Patients exhibiting high n-STAT3 expression (H-score ≥ 70) demonstrated significantly better 10- and 20-year recurrence-free survival (RFS) than those with low n-STAT3 expression. Importantly, n-STAT3 expression provides prognostic information that may complement the ATA risk stratification system [5,6]. Low n-STAT3 scores were significantly enriched in tumors harboring *TERT* promoter mutations, which are known drivers of dedifferentiation and poor clinical outcomes [72,73,74]. This inverse association signifies that low n-STAT3 expression marks a subset of biologically aggressive *TERT*-mutant tumors that have escaped STAT3-mediated growth restraint, underscoring the necessity of interpreting STAT3 activity within the tumor’s specific molecular context.

### 3.4. Crosstalk with Thyroid Hormone Receptors (TR)

Thyroid hormone receptors, particularly TRβ, function as potent tumor suppressors in various cancer types, including thyroid carcinoma. TRβ exerts its suppressive effects by downregulating major oncogenic networks, including the PI3K-AKT and JAK-STAT signaling pathways [75,76]. Loss or mutation of the *THRB* gene, which encodes TRβ, is frequently observed in thyroid cancers and removes this critical inhibitory crosstalk [77]. In ATC, the therapeutic re-expression of endogenous TRβ using demethylating agents, such as decitabine, effectively curbs cancer stem cell (CSC) activity, halts cell proliferation, and enhances apoptotic cell death, further highlighting the importance of the TRβ-STAT axis in maintaining cellular homeostasis and preventing dedifferentiation [78,79].

## 4. Roles of Other STAT Family Proteins in Thyroid Cancer

Although STAT3 is the most extensively studied member of the STAT family in thyroid cancer, other STAT proteins, namely, STAT1, STAT2, STAT4, STAT5A/B, and STAT6, exhibit multifaceted context-dependent roles spanning normal thyroid physiology, autoimmune thyroid diseases, and tumor progression (Table 1).

### 4.1. STAT1

STAT1 plays a highly complex, context-dependent, and dual role in thyroid neoplasia. Traditionally recognized as a tumor suppressor, STAT1 DNA-binding activity is significantly lower in PTC tumors than in surrounding normal thyroid tissue, and this activity is inversely correlated with tumor size [80]. Furthermore, PTCs harboring *BRAF* p.V600E mutations show lower STAT1 activity than wild-type tumors, suggesting that STAT1 suppression is a biomarker of PTC aggressiveness. Crucially, STAT1 has recently been identified to regulate thyroid differentiation by directly activating the transcription of key differentiation genes, including *TSHR*, *SLC26A4* (Pendrin), *SLC5A5* (NIS), *TPO*, and *PAX8* [81]. In ATC, the RNA-binding protein IGF2BP2 binds to *m^6^A*-modified *STAT1* mRNA and accelerates its decay, leading to the loss of differentiation markers and driving profound dedifferentiation [81]. STAT1 also binds to the promoter region of the lncRNA *LINP1* to promote cell proliferation and inhibit apoptosis in PTC [82].

Paradoxically, STAT1 can function as an oncogenic driver in specific PTC contexts. STAT1 can directly bind to the *TCTN3* promoter to drive its transcription, thereby accelerating cell cycle progression, migration, and invasion [83]. Additionally, in *BRAF* p.V600E-mutant thyroid cancer, the acquisition of resistance to BRAF inhibitors (such as vemurafenib) is strongly associated with rapid compensatory activation of the JAK/STAT1 pathway [84]. This activation results in massive upregulation of interferon-stimulated genes, including *IRF1* and *ICAM1*, which mediate tumor cell survival and escape from targeted therapy.

### 4.2. STAT5A and STAT5B

Although encoded by separate genes and sharing over 90% sequence identity, STAT5A and STAT5B exhibit both redundant and distinct functional activities in normal physiology and in carcinogenesis. Classically, STAT5A is the primary mediator of prolactin (PRL) signaling, whereas STAT5B is critical for mediating growth hormone (GH) responses. Despite these distinct physiological roles, hyperactivation of either isoform in the context of malignancy generally acts as an oncogenic driver that upregulates pro-survival and pro-proliferative factors, such as Cyclin D1 and Bcl-xL [77,85].

Physiologically, STAT5 activity is tightly regulated and suppressed by crosstalk with TRβ [86]. Specifically, TRβ1 physically interacts with STAT5A in the cytoplasm. While this physical interaction can promote STAT5A nuclear translocation independently of triiodothyronine (T3), the presence of T3 causes TRβ1/RXR heterodimers to inhibit PRL-induced STAT5A transactivation. This provides a critical counter-regulatory mechanism by which thyroid hormone signaling acts as a physiological brake on the STAT5-mediated gene expression. However, the loss, downregulation, or mutation of TRβ, which is frequently observed in FTC and ATC, removes this vital inhibitory brake [76,77,87,88]. The release of this suppression unleashes constitutive and unchecked JAK2/STAT5 signaling, which powerfully drives thyroid tumor cell proliferation and impairs apoptosis.

### 4.3. STAT6 and STAT4

STAT4 and STAT6 play critical and opposing roles in autoimmune thyroid diseases, which often create an inflammatory background for thyroid lesions. Experimental models have definitively established that STAT6-dependent Th2 immunity is essential for the development of Graves’ disease and the production of stimulatory anti-TSH receptor antibodies [89,90]. Conversely, STAT4 (mediating Th1 immunity) is dispensable for Graves’ disease, and its deficiency may paradoxically worsen Th2-driven hyperthyroidism [91].

In aggressive thyroid cancer, STAT6 serves as a powerful survival factor. The autocrine production of IL-4 and IL-10 by thyroid cancer cells continually activates the JAK1/STAT6 pathway (alongside STAT3) [68]. This activation strongly upregulates anti-apoptotic proteins (such as Bcl-xL and c-Flip), profoundly conferring resistance to conventional chemotherapy in poorly differentiated and anaplastic carcinomas [92]. Re-expression of negative regulators, such as SOCS1 or SOCS3, completely abolishes STAT6 phosphorylation and sensitizes these aggressive cells to chemotherapeutic drugs [53]. Clinically, cytoplasmic and nuclear STAT6 are upregulated in thyroid carcinoma compared to adenoma, correlating with immune infiltration (including PD-L1/CTLA-4 expression), and predict better overall survival, positioning them as potential immunotherapy response biomarkers [93,94]. Additionally, strong nuclear STAT6 immunohistochemical staining is highly sensitive and specific for the NAB2-STAT6 fusion, making it a critical diagnostic tool for differentiating solitary fibrous tumors in the perithyroid space from primary thyroid malignancies [95].

### 4.4. STAT2

STAT2 primarily mediates type I interferon (IFN-α/β) signaling as part of the ISGF3 complex [96]. In the thyroid, *STAT2* mRNA expression is notably increased in autoimmune thyroiditis-related hypothyroidism, suggesting a contribution to an interferon-driven inflammatory milieu [97]. In the ATC TME, STAT2 acts to amplify the Type I IFN/ISGF3 axis alongside STAT1, although its intrinsic mechanistic role in thyroid cancer cells requires further elucidation [98].

**Table 1:** Summary of the context-dependent roles and clinical implications of STAT family proteins in thyroid cancer.

STAT Member	Primary Roles in Thyroid Cancer & Pathology	Underlying Mechanisms & Clinical Implications
STAT1	Master regulator of differentiation; exhibits a dual role (tumor suppressor vs. oncogenic driver) and mediates drug resistance.	Differentiation: Directly activates key thyroid genes (e.g., *PAX8* and *NIS*) [81]. Oncogenic: Accelerates PTC progression via the STAT1-TCTN3 axis [83]. Drug Resistance: Mediates BRAF inhibitor resistance by rapidly upregulating survival genes (*IRF1* and *ICAM1*) [84].
STAT2	Tumor microenvironment (TME) modulator; involved in autoimmune thyroid conditions.	TME: Amplifies Type I IFN/ISGF3 signaling in ATC TME [98]. Clinical: mRNA levels are elevated in autoimmune thyroiditis-related hypothyroidism, shaping the inflammatory milieu [97].
STAT3	A complex “double-edged sword” that acts as both a potent oncogene and a paradoxical tumor suppressor.	Oncogenic: Hyperactivated in advanced PTC and ATC to drive EMT, angiogenesis, cancer stem cell maintenance, and chemo/BRAF-inhibitor resistance [69,70]. Tumor Suppressor: Restrains the Warburg effect (aerobic glycolysis) and upregulates the tumor suppressor *IGFBP7* [11]. Clinical: High nuclear STAT3 levels independently predict favorable recurrence-free survival in PTC and are inversely correlated with *TERT* mutations [71].
STAT4	Immune microenvironment regulator (Th1 polarization); critical in autoimmune backgrounds.	Immune Regulation: Drives Th1 polarization, potentially affecting PD-1 therapy responsiveness [91]. Autoimmunity: Paradoxically, STAT4 deficiency exacerbates Th2-driven Grave’s disease by removing Th1 counterregulation [91].
STAT5A/B	Promotes tumor cell proliferation and survival; modulated by Thyroid Hormone Receptor β (TRβ).	Proliferation/survival: Loss of TRβ in FTC/ATC results in constitutive activation of JAK2/STAT5 signaling, upregulation of Cyclin D1 and Bcl-xL, and impaired apoptosis [77,85].
STAT6	Confers profound chemoresistance; major driver of Th2-mediated autoimmune responses.	Chemoresistance: Constitutively activated by autocrine IL-4/IL-10 loops, powerfully upregulating anti-apoptotic proteins (Bcl-xL, c-Flip) in aggressive cancers [92]. Autoimmunity: Essential driver of Th2-mediated Grave’s disease [66,67]. Clinical: High expression is correlated with PD-L1 immune infiltration [93,94]. Diagnostic marker for NAB2-STAT6 fusions in perithyroidal solitary fibrous tumors [95].

## 5. STAT3 as a Therapeutic Target

Given its profound involvement in tumor progression, EMT, and acquired drug resistance, the JAK/STAT pathway is a highly promising therapeutic target for aggressive thyroid cancer (Table 2). However, the historical perception of STAT3 as an “undruggable” transcription factor, combined with its dual tumor-suppressive and oncogenic functions, necessitates advanced pharmacological strategies and careful stratification of patients.

### 5.1. Precision Medicine and Patient Stratification

A critical prerequisite for implementing STAT3-targeted therapies is the recognition of its “double-edged sword” nature. In early- or intermediate-stage PTC, n-STAT3 functions as a tumor suppressor. Thus, indiscriminately applying STAT3 inhibitors to these patients could inadvertently accelerate tumor progression [14]. Therefore, targeting STAT3 is primarily justified for advanced, radioiodine-refractory (RAIR) thyroid carcinoma or ATC, where aberrant STAT3 activity explicitly drives stemness, dedifferentiation, and drug resistance. Lessons from large-scale clinical trials of other cancers underscore this need: the STAT3/cancer stemness inhibitor napabucasin (BBI608) failed to improve survival in unselected colorectal cancer cohorts but significantly improved overall survival exclusively in the pSTAT3-positive patient subgroup [99]. Clinical application in thyroid cancer must similarly integrate robust biomarkers, such as n-STAT3 H-scoring and mutational profiling, to identify appropriate patient populations.

### 5.2. Direct Small-Molecule STAT3 Inhibitors

Direct inhibition of STAT3 has been challenging. Early-generation oral STAT3 inhibitors (e.g., OPB-51602 and OPB-31121) achieved target suppression but were severely limited by dose-limiting toxicities, particularly severe lactic acidosis and early onset peripheral neuropathy [100,101]. These toxicities are linked to the unintended disruption of STAT3’s non-canonical function in mitoSTAT3, which supports the electron transport chain [102].

Recent breakthroughs have addressed these limitations. TTI-101, a first-in-class, orally bioavailable small molecule, competitively binds to the SH2 domain of STAT3, preventing its phosphorylation, dimerization, and nuclear translocation without impairing its mitochondrial function. In a Phase I trial (NCT03195699) involving patients with advanced solid tumors, TTI-101 was exceptionally well tolerated (with no dose-limiting toxicities) and demonstrated a 12% confirmed partial response (cPR) rate [103]. Notably, in patients with advanced hepatocellular carcinoma, the cPR rate was 20%, particularly in tumors harboring mutant p53, which heavily relies on STAT3 for survival. Preclinical thyroid cancer models mirror these successes; the specific STAT3 inhibitor Stattic radically suppressed sphere-forming capabilities (cancer stem cells) and enhanced tumor cell apoptosis in ATC xenografts [104].

### 5.3. Targeting Upstream Kinases: JAK and IL-6 Inhibition

An alternative to direct STAT3 inhibition is the blockade of its upstream activators. JAK inhibitors (e.g., ruxolitinib, AZD1480, and AG490) effectively abrogate STAT3 phosphorylation. For instance, ruxolitinib significantly decreases the expression of downstream survival targets (e.g., *BCL2L1* and *MYC*) and suppresses cellular proliferation in *RAS*-positive ATC [9,105]. Similarly, natural diterpenoids, such as oridonin, suppress metastatic phenotypes and angiogenesis by downregulating JAK2/STAT3 signaling [62]. AZD1480 effectively blocks STAT3 signaling and inhibits the proliferation and tumor growth of RET-activated thyroid cancer cell lines via STAT3 inhibition [106,107].

However, the clinical utility of upstream JAK inhibitors has critical limitations. Primarily, they lack specificity (simultaneously inhibiting STAT1, which is crucial for antitumor immunity). Furthermore, these agents fail to abrogate JAK-independent STAT3 activation, which can be directly driven by mutant receptor tyrosine kinases (e.g., EGFR and RET) and non-receptor tyrosine kinases (e.g., Src). In malignancies that are heavily dependent on cytokine-driven feed-forward loops, targeting the cytokine receptor provides a viable alternative strategy. For instance, in *BRAF* p.V600E-mutated cells, employing the anti-IL-6 receptor antibody tocilizumab effectively disrupts the autocrine IL-6/STAT3 survival loop [14,69]. This targeted receptor blockade significantly improved cell cycle arrest and successfully overcame acquired resistance to BRAF inhibitors.

### 5.4. Next-Generation Approaches: ASOs and PROTACs

To bypass the structural challenges of STAT3, next-generation modalities are currently in early clinical development.

Antisense Oligonucleotides (ASOs), Danvatirsen (AZD9150), selectively binds and degrades STAT3 mRNA. In clinical trials, it has shown single-agent activity against highly refractory lymphomas and solid tumors [108]. Beyond tumor cells, danvatirsen efficiently penetrates the TME to reduce STAT3 levels in immune and stromal cells, thereby reversing immunosuppression [109].

Targeted protein degraders, such as proteolysis-targeting chimeras (PROTACs), including KT-333 and SD-36, recruit E3 ubiquitin ligases to induce complete and durable STAT3 degradation [110,111]. KT-333 has entered Phase I trials for hematological and solid malignancies, representing a potent strategy to permanently abolish both canonical and non-canonical STAT3 functions.

### 5.5. Combination Therapies to Overcome Resistance

Integrating STAT3 inhibitors into combinatorial regimens is essential for overcoming acquired therapeutic resistance and enhancing tumor immunity.

#### 5.5.1. Overcoming BRAF Inhibitor Resistance

Exposure to BRAF inhibitors (e.g., vemurafenib) rapidly induces massive compensatory upregulation of the IL-6/JAK2/STAT3 and STAT1 signaling axes in thyroid cancer. Dual blockade, combining vemurafenib with either JAK2 inhibitors (AZD1480) or direct STAT3 inhibitors (Stattic), synergistically collapses this resistance mechanism, inducing profound cell cycle arrest and apoptosis in refractory models [60,69,112].

#### 5.5.2. Synergy with Immune Checkpoint Inhibitors (PD-1/PD-L1 Blockade)

In advanced thyroid cancers, such as PDTC and ATC, tumor cells frequently evade immune surveillance through the upregulation of programmed cell death ligand 1 (PD-L1) and polarization of tumor-associated macrophages into the immunosuppressive M2 phenotype—processes that are directly driven by aberrant STAT3 activation [113,114]. While monotherapy with immune checkpoint inhibitors (ICIs), such as the anti-PD-1 antibody pembrolizumab, has shown limited overall response rates in unselected advanced thyroid cancer cohorts (e.g., KEYNOTE-028 and KEYNOTE-158 trials), combining ICIs with JAK/STAT pathway inhibitors provides a strong biological rationale for overcoming primary resistance [114,115,116]. Inhibiting STAT3 downregulates PD-L1, reduces regulatory T cell (Treg) and myeloid-derived suppressor cell (MDSC) infiltration, and promotes cytotoxic CD8+ T-cell activity, effectively converting an immunosuppressive “cold” TME into a “hot,” immune-responsive TME. This combinatorial strategy is currently being investigated in other solid tumors. For instance, the direct STAT3 inhibitor TTI-101 is being evaluated in Phase Ib/II trials in combination with pembrolizumab or atezolizumab (NCT05440708), and the STAT3 antisense oligonucleotide danvatirsen combined with durvalumab (anti-PD-L1) has demonstrated promising clinical activity in advanced solid malignancies [114]. Translating these combinations to radioiodine-refractory and anaplastic thyroid cancers is critical for improving the efficacy of immunotherapy.

**Table 2:** Overview of STAT3-targeted therapeutic strategies and their applications in thyroid cancer and other solid tumors.

Therapeutic Class	Representative Agents	Mechanism of Action	Development Stage & Application in Thyroid Cancer
Direct Small-Molecule Inhibitors (SH2 Domain)	TTI-101, Stattic, S3I-201	Competitively binds the SH2 domain, preventing STAT3 phosphorylation, dimerization, and nuclear translocation while sparing non-canonical mitochondrial function.	TTI-101: Orally bioavailable. Phase Ib/II studies of combination therapy with PD-L1 inhibitors for solid tumors (e.g., HCC) [103]. Stattic: Preclinically enhances BRAF inhibitor efficacy in ATC xenografts [104]. S3I-201: Preclinically suppresses tumor growth and metastasis in thyroid cancer cells [65,66].
Upstream Kinase Inhibitors (JAK Inhibitors)	Ruxolitinib, AZD1480, AG490	Blocks upstream Janus kinases (JAK1/2) to abrogate the phosphorylation of STAT3 and other STAT family members.	AZD1480: Preclinically suppresses tumor growth [84,85] and overcomes drug resistance to BRAF inhibitors [69,84]. Ruxolitinib: Decreases downstream survival molecules, such as BCL2L1 and MYC, reducing cellular proliferation in *RAS*-positive ATC [105].
Cytokine Receptor Blockade	Tocilizumab (anti-IL-6R)	Disrupts the autocrine/paracrine IL-6/STAT3 feed-forward survival loop, which is often upregulated in response to targeted therapies.	Preclinically, combining tocilizumab with BRAF inhibitors improves cell cycle arrest and overcomes acquired therapeutic resistance to BRAF inhibitors [107].
Natural Compounds	Oridonin	Natural diterpenoid that suppresses the JAK2/STAT3 signaling axis.	Preclinically, inhibits EMT and angiogenesis in thyroid cancer cells [62].
Antisense Oligonucleotides (ASOs)	Danvatirsen (AZD9150)	Selectively binds and degrades *STAT3* mRNA.	Phase II clinical trials have shown promising activity in reversing immunosuppression when combined with immune checkpoint inhibitors in advanced solid tumors [108].
Targeted Protein Degraders (PROTACs)	KT-333, SD-36, SD-436	Recruits E3 ubiquitin ligases to induce complete and durable degradation of the entire STAT3 protein.	KT-333: Phase Ia/Ib clinical trials for hematological and solid malignancies [110,111].
Combinations with Immune Checkpoint Inhibitors	STAT3i + Pembrolizumab or Atezolizumab	Induces STAT3 downregulation leading to PD-L1 reduction and activation of cancer immunity.	Combination therapy with PD-L1 inhibitors and TTI-101 is currently in Phase Ib/II clinical trials [103].
Combinations with STING Agonists	STAT3i + TAK-676 or c-diAM(PS)	The cGAS-STING pathway triggers a negative feedback loop via IL-6/STAT3, which dampens innate immunity.	Preclinically, STAT3 inhibition and STING activation synergistically induce an immune-responsive microenvironment [117].

#### 5.5.3. Synergy with STING Agonists

The cGAS-STING pathway is critical for innate antitumor immunity; however, its activation paradoxically triggers a negative feedback loop via IL-6/STAT3 signaling, dampening the immune response [16]. Inhibiting STAT3 severs this immunosuppressive feedback, dramatically enhancing the intensity and duration of STING agonists [118]. Preclinical models have shown that combined STAT3 inhibition and STING activation remodels the TME by increasing cytotoxic CD8+ T cells while depleting regulatory T cells and myeloid-derived suppressor cells (MDSCs) [119]. This highlights a powerful rationale for combining agents such as TTI-101 with STING agonists or immune checkpoint inhibitors to convert “cold” aggressive thyroid tumors into immune-responsive tumors [117].

## 6. Conclusions

The roles of STAT family proteins in thyroid cancer are multifaceted. STAT3 embodies a complex “double-edged sword”. In advanced and RAIR carcinomas, hyperactive STAT3 acts as a potent oncogene that drives invasion, cancer stem cell renewal, and resistance to targeted therapy. Paradoxically, in differentiated thyroid cancers, it functions as a robust tumor suppressor, restraining the Warburg effect and limiting tumor expansion. Clinically, high nuclear STAT3 expression serves as an independent, favorable prognostic biomarker that is inversely correlated with aggressive *TERT* promoter mutations, providing a biological modifier to refine the 2025 ATA risk stratification system.

Other STAT family proteins are also involved in thyroid cancer proliferation and treatment resistance by directly or indirectly influencing the signaling pathways within cancer cells or by modulating the TME, especially the immune environment.

While next-generation agents have overcome STAT3’s historic “undruggability,” indiscriminate inhibition risks abrogating its innate tumor-suppressive functions. Therefore, STAT-targeted therapies require meticulous biomarker-guided precision medicine. Future therapeutic frontiers lie in rational combinatorial strategies, such as coupling STAT3 inhibitors with immune checkpoint inhibitors or STING agonists to reverse immunosuppression and coupling STAT3 inhibitors with drugs targeting STAT1 to induce redifferentiation and restore radioiodine sensitivity in RAIR thyroid malignancies.

## Data Availability

No new data were created or analyzed in this study.

## Abbreviations

The following abbreviations are used in this manuscript:

ASO	Antisense Oligonucleotide
ATA	American Thyroid Association
ATC	Anaplastic Thyroid Carcinoma
CCD	Coiled-Coil Domain
cGAS	Cyclic GMP-AMP Synthase
DBD	DNA-Binding Domain
DNMT1	DNA Methyltransferase 1
DTC	Differentiated Thyroid Carcinoma
EMT	Epithelial–Mesenchymal Transition
EZH2	Enhancer of Zeste Homolog 2
FTC	Follicular Thyroid Carcinoma
GAS	Gamma-Activated Site
HAT	Histone acetyltransferase
HCC	Hepatocellular Carcinoma
HDAC	Histone Deacetylase
HIF1α	Hypoxia-Inducible Factor 1-alpha
HP1	Heterochromatin Protein 1
ICI	Immune Checkpoint Inhibitor
IFN	Interferon
IGFBP7	Insulin-like Growth Factor Binding Protein 7
IL	Interleukin (e.g., IL-4, IL-6, IL-10)
ISGF3	Interferon-Stimulated Gene Factor 3
ISRE	Interferon-Stimulated Response Element
JAK	Janus Kinase
KIR	Kinase Inhibitory Region
LD	Linker Domain
lncRNA	Long non-coding RNA
m^6^A	N6-methyladenosine
MDSC	Myeloid-Derived Suppressor Cell
mitoSTAT3	Mitochondrial STAT3
n-STAT3	Nuclear STAT3
NF-κB	Nuclear Factor kappa-light-chain-enhancer of activated B cell
NTD	Amino-Terminal Domain
OXPHOS	Oxidative Phosphorylation
PD-1	Programmed Cell Death Protein 1
PD-L1	Programmed Cell Death Ligand 1
PDTC	Poorly Differentiated Thyroid Carcinoma
PIAS	Protein Inhibitor of Activated STAT
PROTAC	Proteolysis Targeting Chimera
pSTAT3	Phosphorylated STAT3
PTC	Papillary Thyroid Carcinoma
PTM	Post-Translational modification
PTP	Protein Tyrosine Phosphatase
RAIR	Radioiodine-Refractory
RFS	Recurrence-Free Survival
SENP3	SUMO Specific Peptidase 3
SET9	SET Domain Containing 9
SH2	Src Homology 2
SOCS	Suppressor of Cytokine Signaling
STAT	Signal Transducer and Activator of Transcription
STING	Stimulator of Interferon Genes
TAD	Transactivation Domain
TME	Tumor Microenvironment
TR	Thyroid Hormone Receptor (e.g., TRβ)
Treg	Regulatory T Cell
Ub	Ubiquitin
uSTAT3	Unphosphorylated STAT3
